# Time-Dependent Material Properties of Aging Biomolecular Condensates from Different Viscoelasticity Measurements in Molecular Dynamics Simulations

**DOI:** 10.1021/acs.jpcb.3c01292

**Published:** 2023-05-17

**Authors:** Andrés R. Tejedor, Rosana Collepardo-Guevara, Jorge Ramírez, Jorge R. Espinosa

**Affiliations:** † Maxwell Centre, Cavendish Laboratory, Department of Physics, University of Cambridge, J. J. Thomson Avenue, Cambridge CB3 0HE, United Kingdom; ‡ Department of Chemical Engineering, Universidad Politécnica de Madrid, José Gutiérrez Abascal 2, 28006 Madrid, Spain; § Yusuf Hamied Department of Chemistry, University of Cambridge, Lensfield Road, Cambridge CB2 1EW, United Kingdom; ∥ Department of Genetics, University of Cambridge, Downing Site, Cambridge CB2 3EH, United Kingdom; ⊥ Departament of Chemical Physics, Faculty of Chemical Sciences, Universidad Complutense de Madrid, 28040 Madrid, Spain

## Abstract

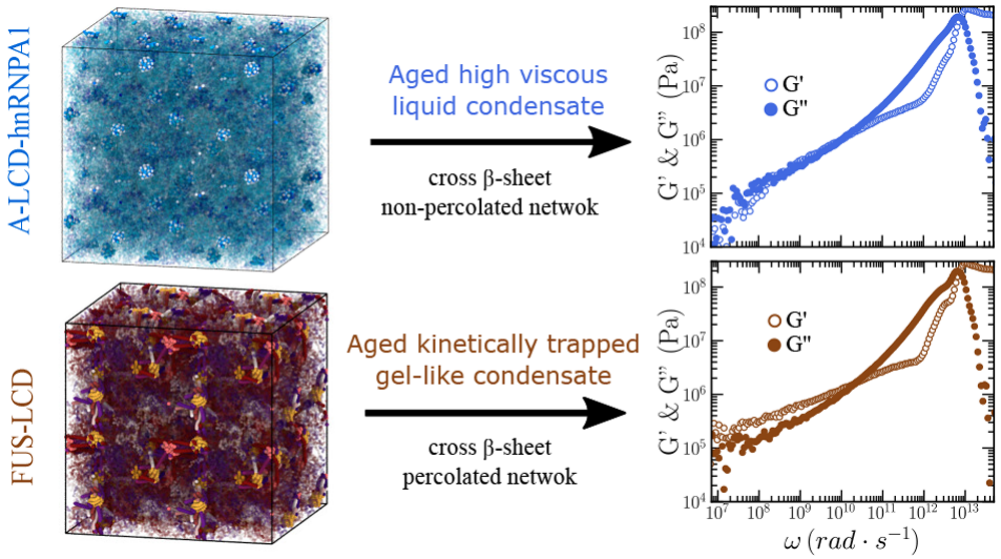

Biomolecular condensates are important contributors to the internal organization of the cell material. While initially described as liquid-like droplets, the term biomolecular condensates is now used to describe a diversity of condensed phase assemblies with material properties extending from low to high viscous liquids, gels, and even glasses. Because the material properties of condensates are determined by the intrinsic behavior of their molecules, characterizing such properties is integral to rationalizing the molecular mechanisms that dictate their functions and roles in health and disease. Here, we apply and compare three distinct computational methods to measure the viscoelasticity of biomolecular condensates in molecular simulations. These methods are the Green–Kubo (GK) relation, the oscillatory shear (OS) technique, and the bead tracking (BT) method. We find that, although all of these methods provide consistent results for the viscosity of the condensates, the GK and OS techniques outperform the BT method in terms of computational efficiency and statistical uncertainty. We thus apply the GK and OS techniques for a set of 12 different protein/RNA systems using a sequence-dependent coarse-grained model. Our results reveal a strong correlation between condensate viscosity and density, as well as with protein/RNA length and the number of stickers vs spacers in the amino acid protein sequence. Moreover, we couple the GK and the OS technique to nonequilibrium molecular dynamics simulations that mimic the progressive liquid-to-gel transition of protein condensates due to the accumulation of interprotein β-sheets. We compare the behavior of three different protein condensates, i.e., those formed by either hnRNPA1, FUS, or TDP-43 proteins, whose liquid-to-gel transitions are associated with the onset of amyotrophic lateral sclerosis and frontotemporal dementia. We find that both the GK and OS techniques successfully predict the transition from functional liquid-like behavior to kinetically arrested states once the network of interprotein β-sheets has percolated through the condensates. Overall, our work provides a comparison of different modeling rheological techniques to assess the viscosity of biomolecular condensates, a critical magnitude that provides information on the behavior of biomolecules inside condensates.

## Introduction

Biomolecular condensates are membraneless assemblies that contribute to the spatiotemporal organization of biomolecules in the cytoplasm and the nucleoplasm.^1−6^ These condensates, mainly formed by multivalent proteins and nucleic acids,^7,8^ actively participate in numerous aspects of the cell function, such as in compartmentalization,^6,9−13^ genome organization,^14−17^ gene expression,^14,18,19^ formation of superenhancers,^20^ cell signaling,^2,21^ or the sequestration of harmful components in the cell^22^ among many others.^23−27^ Biomolecular condensates are thought to form via the process of liquid–liquid phase separation (LLPS), which refers to the physicochemical demixing of a biomolecular mixture into different coexisting liquid phases with different concentrations.^3^ Microscopically, liquid-like behavior within phase-separated condensates originates on weak multivalent attractive interactions that proteins and nucleic acids can establish.^28^ Such weak and transient intermolecular interactions translate into dynamic binding and unbinding, free molecular diffusion within condensates, and facile exchange of species in and out of the condensates.^15,16^ Initially, the liquid-like behavior of the molecules within the condensates was thought to be a defining feature of such systems. However, more recently, the material properties of biomolecular condensates have been recognized as more diverse than initially anticipated, with condensates encompassing low to high viscosity fluids,^29,30^ hydrogels,^31,32^ and even solid-like states.^33,34^

While the liquid-like behavior of the condensates seem to underpin their functions during health,^35,36^ kinetically trapped states are often associated with the proliferation of multiple neurodegenerative disorders,^37^ such as amyotrophic lateral sclerosis (ALS),^32^ Parkinson’s,^38^ Alzheimer’s,^39^ or frontotemporal dementia (FTD), as well as to certain types of cancers^40^ and diabetes.^41^ Several factors that have been proposed as key drivers for condensate liquid-to-gel/solid transitions include altered salt-concentration or temperature,^29,42^ post-translational modifications,^39,43^ protein mutations,^44−46^ and most prominently, protein folding and misfolding events.^13,47−51^ All these factors are expected to favor progressive condensate rigidification by increasing the binding affinity among species, and therefore, slowing down the time scales of interprotein unbinding events.

To characterize the progressive rigidification of condensates that initially display liquid-like behavior and gradually change their material properties into gels or soft glasses, i.e., “maturation”, several experimental techniques including fluorescence recovery after photobleaching (FRAP), green fluorescence protein (GFP) FRAP, fluorescence correlation spectroscopy, or active microrheology have been successfully employed.^12,29,52−55^ Viscoelastic properties such as viscosity (η) have also been measured through passive microrheology techniques; i.e., bead-tracking,^56−62^ in which the trajectory of the beads can be registered and their mean squared displacement (MSD) calculated. Then, the droplet viscosity can be inferred from the diffusion coefficient obtained through the MSD using the Stokes–Einstein relation.^63^ Matured condensates usually exhibit reduced fusion propensities and longer recovery times after photobleaching,^4,52,53,64−69^ which suggest that the diffusion of the molecules within the condensate is significantly reduced. While viscoelastic measurements allow us to identify the gradual transition of functional condensates into pathological aggregates, they are not sufficient on their own to uncover the underlying molecular mechanisms of such transitions. Rationalizing from a microscopic perspective the dysregulation of condensates into pathological aggregates is fundamental to devise effective strategies to prevent condensate age-related diseases^32^ such as neurodegenerative disorders^70^ and some types of cancer.^71^

Computer simulations are a powerful tool to uncover the molecular mechanisms that explain the changes in viscosity within biomolecular condensates over time.^30,50,62,72−75^ From atomistic force fields^76−81^ to coarse-grained (CG) models,^82−91^ including lattice-based simulations^92−94^ and mean-field theory,^95−97^ computer simulations have significantly contributed to elucidating factors behind biomolecular phase-separation such as protein and RNA length,^98−100^ amino acid patterning,^90,101−104^ multivalency,^34,105−108^ conformational flexibility^88,109^ or multicomponent composition.^89,110,111^ Remarkably, coarse-grained models have uncovered the impact of enhancement of interprotein interactions in condensate rigidification,^50,73^ as well as the formation of kinetically arrested multiphase condensates from single-component droplets.^72,112^ Nevertheless, further insights on the molecular driving forces behind condensate maturation, for instance, those triggered by interprotein disordered-to-order structural transitions,^49,74,113^ amino acid sequence mutations^114^ or relevant variations on the applied thermodynamic conditions,^115^ are urgently needed.

In this work, we apply three different computational methods to evaluate the viscoelastic behavior of biomolecular condensates formed by proteins and RNA. These methods are the Green–Kubo (GK) relation,^116,117^ the oscillatory shear (OS) technique,^118,119^ and passive microrheology bead tracking (BT).^29,120−122^ Although these techniques are well-known in the field of polymer physics,^116,123^ here we test them for the first time in the context of biomolecular condensates and progressive condensate maturation. First, we assess their performance in terms of statistical uncertainty, computational efficiency, and implementation cost using a simple intrinsically disordered protein (IDP) coarse-grained model. Importantly, we find that the three methods provide consistent results for condensate viscosities under different conditions. However, the performance in terms of computational efficiency and statistical error is significantly poorer for the BT technique. Then, we apply the GK and OS techniques for determining the droplet viscosity of a set of 7 different IDPs and 5 peptide/RNA complex coacervates using a sequence-dependent coarse-grained model.^101,124,125^ As expected, in all cases, the agreement between the GK and OS methods to evaluate viscosity is reasonable. Furthermore, we identify a clear correlation between the condensate viscosity and IDP length, as well as with the number of stickers vs spacers^126^ in the amino-acid sequence when viscosity is measured at a constant ratio of temperature (*T*) over the critical temperature of each system (*T*_*c*_^′^). However, when temperature is kept constant, instead of *T*/*T*_*c*_^′^, the viscosity correlates with the condensate density and critical temperature. Finally, we use the GK and OS techniques to track the progressive maturation of three of the most relevant protein low-complexity domains related to the onset of ALS and FTD, which are the heterogeneous ribonucleoprotein A1 (hnRNPA1),^13,113,127^ fused in sarcoma (FUS),^48^ and the TAR DNA-binding Protein of 43 kDa (TDP-43).^49,128^ We find that both the GK relation and oscillatory shear techniques predict the transition from liquid-like behavior to a gel-like state once the intermolecular network of β-sheets has fully percolated through the condensate. Such percolation of strong β-sheets connections frustrates the long-time self-diffusion of proteins within condensates. Taken together, our study provides an evaluation of modeling rheological techniques to evaluate how changes in the intramolecular behavior of biomolecular condensates over time influence their material properties.

## Results

### An IDP Coarse-Grained Model for Benchmarking Viscosity Calculations through Different Techniques

**Figure 1:**
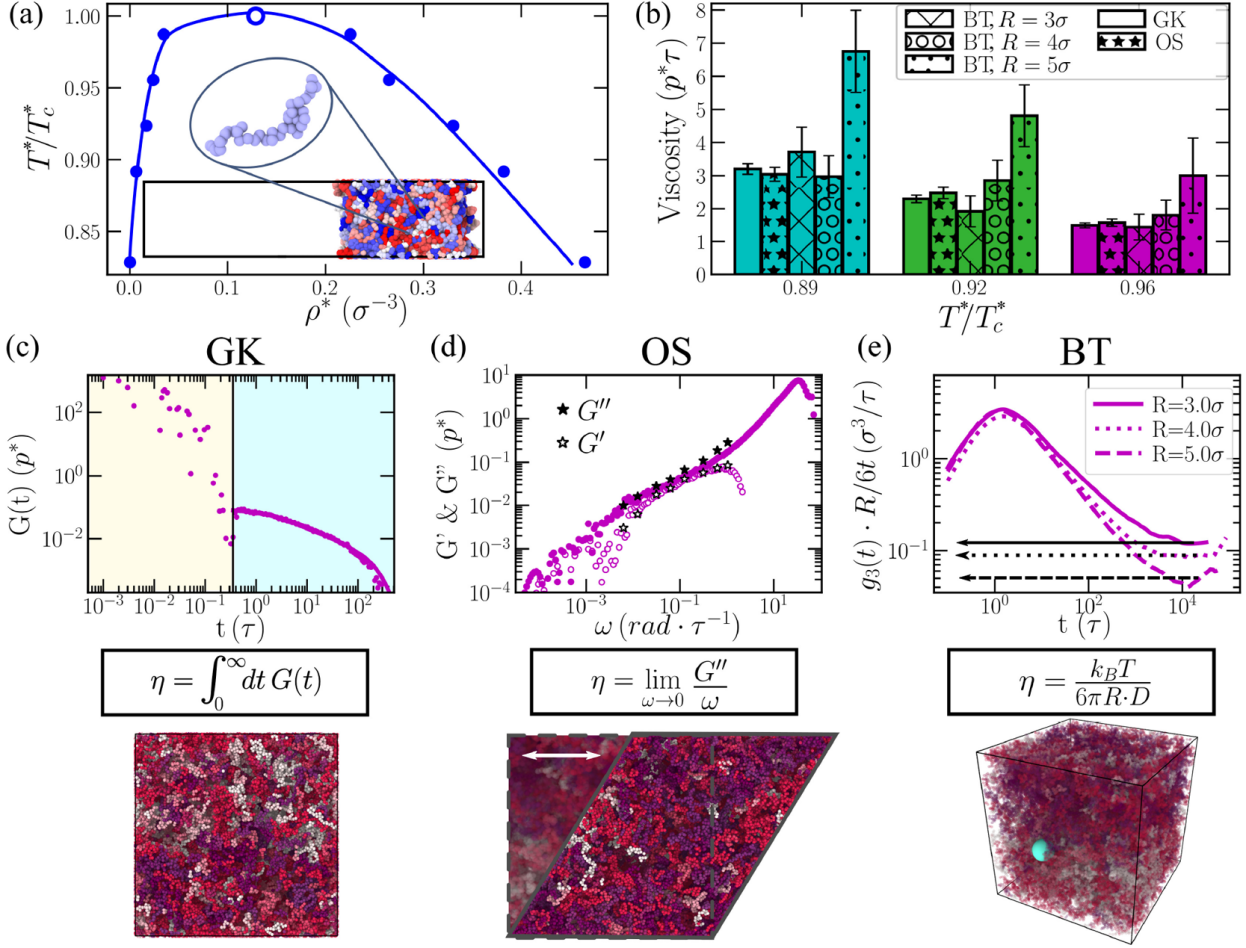
Applied computational methods to evaluate the viscosity in biomolecular condensates. (a) Phase diagram in the T*−ρ* plane for our IDP coarse-grained model using 50-bead chains obtained through Direct Coexistence (DC) simulations.^130^ Filled circles indicate the coexisting densities obtained from DC simulations (the inset shows a phase-separated condensate in a DC simulation), whereas the empty circle accounts for the system critical temperature (*T*_c_^*^ = 3.14) obtained through the law of rectilinear diameters and critical exponents.^131^ (b) Condensate viscosity at different temperatures obtained through GK, OS, and BT calculations as indicated in the legend. For the BT technique, we include results with different probe bead radii as specified in the legend. (c) Top: Shear stress relaxation modulus as a function of time for an IDP condensate at T*/*T*_c_^*^ = 0.96. The vertical black line separates the time scale corresponding to the computed term via numerical integration at short-times and the part evaluated via the Maxwell modes fit at long time scales. Middle: General equation to obtain viscosity through the GK relation. Bottom: IDP condensate simulation box in the canonical ensemble (at the condensate coexisting density) employed to compute *G*(*t*). Different IDPs are colored with different tones (as in (d) and (e) bottom panels). (d) Top: Elastic (*G*′) and viscous (*G*″) moduli as a function of frequency (ω) from OS calculations (empty and filled stars respectively) and from GK (empty and filled purple circles respectively) at T*/*T*_c_^*^ = 0.96. Middle: General equation to obtain viscosity through the OS technique. Bottom: IDP condensate simulation box in the canonical ensemble (at the condensate coexisting density) after applying a shear deformation (γ_*xy*_). (e) Top: Mean-squared displacement (referred as *g*3(*t*)) of an inserted bead within an IDP condensate (T*/*T*_c_^*^ = 0.96) multiplied by *R*/6*t* (referring *R* to the bead radius and *t* to time) as a function of time for beads with different radii as indicated in the legend. The plateau at long time scales (denoted by horizontal lines) shows the value of *R*·*D* (being *D* the diffusion coefficient) at the diffusive regime. Middle: Stokes–Einstein equation for computing viscosity through the BT method. Bottom: IDP condensate simulation box in the canonical ensemble (at the condensate coexisting density) containing a single-bead with a radius of 5σ (green sphere).

Viscosity is a fundamental time-dependent material property of condensates that emerges from the internal friction of proteins within, and thus, changes from the early stages of condensate nucleation to its maturation over time.^29^ Despite its importance, the estimation of the condensate viscosity through computer simulations is not routinely done.^125,129^ Here, we test the validity and computational performance of three different numerical methods to compute viscosity of biomolecular condensates. As an initial test, we employ a simple coarse-grained model for phase-separating IDPs.^99^ In this model, each IDP consists of a flexible polymer of *N* = 50 beads, where each bead represents a group of several amino acids. We mimic the ability of phase-separating model IDPs to establish numerous weak and promiscuous protein–protein interactions at short molecular distances with a short-ranged attractive Lennard-Jones (LJ) potential among nonbonded protein beads:
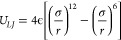
1where σ accounts for the molecular diameter of each bead, *r* is the interbead distance, and ϵ defines the maximum attractive interaction among different beads. The LJ potential is used to broadly approximate the various types of molecular interactions driving LLPS, e.g., hydrophobic, electrostatic, cation−π, and π–π.^28,88^ For computational efficiency, the pair force computed from the gradient of *U*_*LJ*_ is truncated to zero at a cutoff distance of *r*_*c*_ = 3σ so that nonbonded forces act only between pairs of particles with *r* < *r*_*c*_.^99^ To account for the covalent bonds among subsequent groups of amino acids within a given IDP, consecutive beads are joined together with a stiff harmonic potential, *U*_Bond_, of the following form:
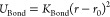
2where *K*_Bond_ controls the stiffness of the bond and *r*_0_ is the equilibrium bond length. The model presents a spring constant *K*_Bond_ = 7.5 × 10^4^ ϵ/σ^2^, and equilibrium bond length corresponding to 1σ. Nonbonded interactions between adjacent beads directly connected are excluded. For computational efficiency, the solvent is modeled implicitly; hence the protein-poor liquid phase corresponds to a vapor phase and the protein-rich liquid phase (or the condensate) to a liquid phase. For this model, we define the following magnitudes in reduced units: temperature as T* = *k*_B_*T*/ϵ, number density as ρ* = (N/V)σ^3^, pressure as p* = *p*σ^3^/ϵ, and reduced time (τ) as ; being ϵ, σ, and *m* equal to 1. The phase diagram in the T*−ρ* plane for our IDP model obtained through Direct Coexistence (DC) simulations^130^ is presented in Figure 1(a). Then, we use cubic boxes emulating the density and temperature at bulk conditions obtained from the phase diagram, to compare three different computational methods to estimate viscosity (these methods as described below). Further details of the simulation system sizes and the DC method are provided in the Supporting Information.

#### Green–Kubo (GK) Relation

To begin, we evaluate the viscosity of a condensate of our IDP model by means of the GK relation. The time-dependent mechanical response of a viscoelastic material when it is subjected to a small shear deformation can be described by the shear stress relaxation modulus (*G*(*t*)).^116^ In the limit of zero deformation, *G*(*t*) can be determined by computing the autocorrelation of any of the off-diagonal components of the pressure tensor at equilibrium:^123,132−134^
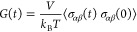
3where σ_αβ_ is an off-diagonal component (αβ) of the stress tensor, *V* is the volume, and the correlation average is taken at equilibrium over all possible time origins. Nevertheless, if the system is isotropic, a more accurate expression of *G*(*t*) can be obtained by using the six independent components of the pressure tensor, as shown in refs (117) and (123).
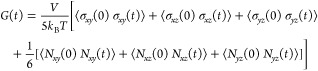
4where *N*_αβ_ = σ_αα_ – σ_ββ_ is the normal stress difference. This correlation can be easily computed on the fly during a simulation, with no significant CPU cost and no need to postprocess the trajectory. For instance, in the LAMMPS Molecular Dynamics (MD) package, this can be done by using the compute ave/correlate/long in the USER-MISC package.^135^

Once the relaxation modulus has been computed, the shear viscosity (η) can be straightforwardly calculated by integrating the shear stress relaxation modulus in time, using one of the GK formulas:^136^
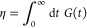
5To avoid the typical noisy nature of the relaxation modulus in the terminal decay region obtained in protein condensate simulations,^74,129^ we follow a particular strategy to estimate the viscosity. While at short time scales *G*(*t*) is smooth and the integral can be computed using numerical integration (eq 5), at longer time scales *G*(*t*) is fitted to a series of Maxwell modes (*G*_*i*_ exp(−*t*/τ_*i*_)) equidistant in logarithmic time,^116,137^ and then the function is integrated analytically. Therefore, viscosity is effectively obtained by adding two different terms
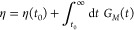
6where η(*t*_0_) corresponds to the computed term at short times, *G*_*M*_(*t*) = ∑^*M*^*G*_*i*_ exp(−*t*/τ_*i*_) is the part evaluated via the Maxwell modes fit at long time-scales, and *t*_0_ is the time that separates both regimes, i.e., black vertical line in Figure 1(c). The division time *t*_0_ is chosen as the time after which all intramolecular oscillations of *G*(*t*) have decayed and the function becomes strictly positive and decays monotonously.

The GK method is exact, within the accuracy of the underlying simulation, and gives the right value of *G*(*t*) in the limit of zero deformation (γ → 0). A similar measurement can be performed experimentally by applying a shear deformation γ and measuring the evolution of the stress response σ_*xy*_(*t*, γ) to determine the shear relaxation modulus as *G*(*t*, γ) = σ_*xy*_(*t*)/γ, in the limit of small γ, where the modulus becomes independent of the deformation amplitude, i.e., the system is in the linear viscoelastic regime (LVE). The GK method has been recently applied by us^74,129^ to evaluate viscosities in phase-separated condensates of RNA-binding proteins, both in the absence and presence of RNA. One of the main advantages of the direct evaluation of *G*(*t*) from simulations is that it provides critical information not only on how the material properties of condensates may change upon maturation but also on how such changes are dictated by different relaxation mechanisms of the proteins that compose them (Figure 1(c)). At short time scales (beige region; Figure 1(c)), the stress relaxation modulus is mostly dependent on the formation and breakage of short-range interactions and on intramolecular reorganization, i.e., intramolecular protein conformational fluctuations, such as bond or angle relaxation modes. In contrast, at long time scales (light blue region; Figure 1(c)), the stress relaxation modulus is mainly dominated by intermolecular interactions, long-range conformational changes, i.e., protein folding/unfolding events, and protein diffusion within the crowded liquid-like environment of the condensate. The calculation of η through the GK method does not depend on the size of the system, apart from the obvious limit to avoid finite size effects. As the system grows in size, the equilibrium value of the shear stress goes to zero, and the fluctuations become smaller. However, the size of the fluctuations of σ_*xy*_ decay with 1/√*V*, and therefore the calculation of *G*(*t*) becomes independent of *V*.

In Figure 1(c), we show the time evolution of *G*(*t*) (purple circles) measured for the IDP model condensate at the coexisting density corresponding to T*/*T*_c_^*^ = 0.96. By numerical integration (beige region) and analytical integration (light blue region) of *G*(*t*), as shown in eq 6, we can obtain the condensate viscosity for different temperatures (Figure 1(b)).

#### Oscillatory Shear (OS) Technique

The second approach that we employ to determine the viscosity of phase-separated condensates is the OS method. In this approach, a sinusoidal strain with angular frequency ω is applied to the condensate in simple shear:
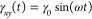
7where γ_*xy*_(*t*) = Δ*L*_*x*_/*L*_*y*_(*t*) represents the shear deformation applied to the simulation box in the *x* direction relative to the box dimension *L*_*y*_, and γ_0_ is the amplitude of the imposed deformation (Figure 1(d)). Please note that the box is cubic so that *L*_*x*_ = *L*_*y*_ = *L*_*z*_ ≡ *L*. This type of deformation can be easily applied to the simulation box by using the “fix deform” command of the LAMMPS package, with the option “wiggle”. If the biomolecular condensate lies within the linear viscoelastic regime, then the stress response will be
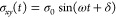
8where σ_0_ refers to the amplitude of the response and δ to the phase shift angle. Within the LVE regime, the ratio σ_0_/γ_0_ is constant and the shear response presents a sinusoidal shape. To determine the optimal γ_0_, an amplitude sweep is needed to ensure that the shear deformation is within the LVE regime but also that the stress response signal with such deformation is detectable (see the Supporting Information for further technical details). Once an amplitude within the LVE regime has been selected (γ_0_; which in our simulations is usually 0.6*L*, where *L* is the size of the initial cubic box), we perform a frequency sweep (avoiding high frequencies to prevent overheating; please note that the maximum frequency should be smaller than the inverse of the characteristic relaxation time of the thermostat), and we measure the transient stress tensor response in the direction of the oscillatory shear (σ_*xy*_(*t*)). Then, by fitting the stress response for each frequency to eq 8 (after 20 periods of sampling), we can calculate the frequency dependent values of σ_0_ and δ. Then, the complex modulus G*(ω) = *G*′(ω) + *i*·*G*″(ω), where *G*′ is the elastic or storage modulus, and *G*″ is the viscous or loss modulus, can be obtained through the following expressions:
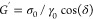
9and
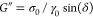
10By means of the OS technique, the viscosity can be estimated in the limit of^116^
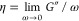
11For computing the viscosity of a liquid in this regime (where *G*″ ∝ ω and *G*′ ∝ ω^2^),^116^ long simulations using large amplitudes are required, so that the stress response is higher than the fluctuations of the system. Furthermore, from the representation of *G*′and *G*″ as a function of ω, the viscoelastic behavior of the system can be inferred. If *G*′ > *G*″, then elasticity dominates over flow, and hence the system exhibits solid-like behavior. On the contrary, the viscoelastic response of a liquid is markedly different. The terminal response of a liquid condensate is dominated by the loss modulus because the stress is nearly in phase with the shear rate (the time derivative of the applied shear deformation γ_*xy*_(*t*)), and hence *G*″ is higher than *G*′ at low frequencies. Although the OS technique has been experimentally applied to numerous soft matter and polymeric systems^118,119^ (some of them including even chocolate^138^ or mozzarella),^139^ its application to protein condensates has been much more limited,^29,140^ mainly due to sample size requirements (bulk rheology measurements need, at least, volumes of the order of milliliters). In computer simulations, the OS technique has been mainly employed to characterize polymeric systems.^116,122,141−143^

In Figure 1(d), we show the values for *G*′ (empty stars) and *G*″ (filled stars) as a function of the applied frequency for a condensate of our IDP coarse-grained model using the OS technique. As can be seen, excellent agreement is obtained with the results from the GK method for *G*′ (purple empty circles) and *G*″ (purple filled circles). Furthermore, when viscosity is estimated through OS by means of eq 11, a good agreement is also found with the predictions of the GK method for IDP condensates at different temperatures (Figure 1(b)). The complex modulus G* can also be obtained from *G*(*t*) by applying a Fourier transform.^116^

#### Bead Tracking (BT) Method

The BT method is a passive microrheology technique widely used in experiments to determine the viscosity of a given material.^116,144^ For biomolecular condensates, this is the technique that has been mainly used to measure experimentally the viscosity of *in vitro* phase-separated droplets displaying both gel-like^29,120,121^ and liquid-like behavior.^58,60,61,140,145^ The idea behind this method is as simple as introducing passive probe spherical beads (with a typical radius, *R*, of the order of hundreds of nanometers),^144^ and measure the mean squared displacement (MSD(t) = ⟨(**r**(*t*) – **r**(0))^2^⟩) of such beads, from which the diffusion coefficient (*D*) of the bead can be calculated as^146,147^
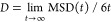
12where the limit indicates the time when the diffusive regime is attained. Importantly, the bead size needs to be larger than the characteristic mesh size of the system. Otherwise, the probe would move freely without experiencing the force of network strands or entanglements.^116^ Then, using the Stokes–Einstein relation,^148^ the viscosity of the medium can be calculated:
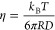
13where *k*_B_ refers to the Boltzmann constant and *T* to the system temperature. This method can also be used to obtain the full frequency-dependent complex modulus,^57^ although here we only focus in the low-frequency Fickian limit. The BT technique is highly suitable for characterizing the viscoelastic properties of biological systems, such as biomolecular condensates,^29,58,60,61,120,121,145^ as it can be performed in volumes of the order of μL. Importantly, microrheology BT can also be performed *in vivo* by tracking the motion of micrometre-sized beads (or even smaller beads^149^) inserted inside cells, as performed in the cytoplasm of developing *Caenorhabditis elegans* embryos.^150^ Although the microrheology bead tracking can be active, i.e., when the particle is moved in the medium by means of optical tweezers or magnetic forces,^29^ here we focus on passive BT where only thermal energy drives the probe particle across the medium exerting minimal deformation, and the motion of the particle is related to the mechanical properties of the medium.

In our simulations, we perform passive single-particle bead tracking to calculate the viscosity of the condensates via the Stokes–Einstein relation (eq 13). The probe particles are modeled with an Ashbaugh–Hatch potential^151^ of the following form:
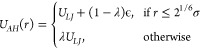
14where *U*_*LJ*_ refers to the standard LJ potential presented in eq 1, ϵ refers to the LJ potential depth (set to ϵ = 4 for the probe bead to ensure no-slip boundary conditions), and λ is a scaling factor that modulates the degree of attraction between probe beads and IDPs (where λ = 0 establishes a purely repulsive interaction and λ = 1 a standard LJ interaction). The mass of the probe bead is set to *m* = 1, and the cutoff distance for the *U*_*AH*_ interaction is 3 times the probe bead molecular diameter. We explore bead particles with radii of 3, 4, and 5 σ (referring σ to the molecular diameter of the residue beads in our IDP coarse-grained model). For both bead–bead self-interactions and bead–IDP cross-interactions, we set a value of λ equal to zero. In this limit, the Ashbaugh–Hatch potential is identical to a Weeks–Chandler–Andersen^152^ potential; hence, the inserted probe beads act as a pseudohard-sphere particle with the surrounding media.^153^ Please note that despite self-interactions among probe beads being set to be purely repulsive, we only study this technique using a single probe bead (see the Supporting Information for further technical details).

Following previous work on bead tracking simulations for polymeric systems,^122^ we introduce a probe bead (with radii of 3, 4, and 5 σ) within phase-separated condensates under bulk conditions (Figure 1(e) bottom panel). By plotting the mean squared displacement (*g*_3_(*t*) ≡ MSD(*t*)) multiplied by *R*/6*t* as a function of time, we can identify the time scale at which the diffusive regime is attained, i.e., when the function reaches a plateau. Subsequently, we introduce the value of such plateau (*R*·*D*) in the Stokes–Einstein equation to obtain η (eq 13). In Figure 1(e; top panel), we depict by black horizontal lines the value of *R*·*D* on the plateau for different bead radii at T*/*T*_c_^*^ = 0.96. Although the values of *R*·*D* depend on the bead radius, the BT method predicts the same viscosity within the uncertainty as the GK and OS methods at T*/*T*_c_^*^ = 0.96 (Figure 1(b)). Nevertheless, such agreement between BT, OS, and GK methods to predict η is only observed at lower temperatures when probe beads of *R* = 3σ and 4σ are introduced within the condensates (Figure 1(b)). The need for smaller beads stems from the much longer time scales required to reach the diffusive regime at low temperatures with very large beads, i.e., *R* = 5σ or 6σ; see Figure S1, which entails a huge computational effort to observe a smooth plateau from which a reliable value of η can be obtained (as it easily occurs for all bead sizes at high temperatures). Moreover, since the probe bead size must be significantly larger than the characteristic mesh size of the system,^116,122^ lowering the bead size below 3σ to increase its diffusion would lead to an underestimate of the condensate viscosity (as shown in Figure S1). Hence, we note that the BT method can only be safely applied to low viscous condensates (or at high temperatures) where the large sampling of the bead trajectory guarantees consistent results independently of the inserted bead size (Figure 1(e); purple bars). In fact, for the three different methods, the uncertainty associated with the calculation becomes lower as we increase temperature, because the protein mobility within the condensate increases due to the higher temperature and lower condensate density (Figure 1(a,b)).

Therefore, despite being conceptually a straightforward approach, the bead tracking method requires long simulation time scales to ensure that the mean squared displacement of the inserted beads is properly sampled up to the Fickian regime. The computational efficiency of the bead tracking method, and thus its associated statistical error, is significantly hampered by the requirements to include only one bead in the simulation box (or at least the concentration of beads must be low enough to guarantee that beads do not interact with each other) and to use probe beads that are larger than the characteristic mesh size. However, it can still provide reasonable estimations of η as shown in Figure 1(b) under certain conditions, i.e., within low viscous media.

### Condensate Viscosity Is Fundamentally Determined by Protein/RNA Length, Stickers Abundance, and Condensate Density

Once the different advantages and drawbacks of the GK, OS, and BT methods have been discussed in determining the viscosity of condensates, we move away from generic proteins and now explore the dependence of the viscosity of 12 different phase-separated protein/RNA condensates using a sequence-dependent coarse-grained model. We compare the changes in viscosity among these systems by focusing on the protein length, amino acid sticker abundance, condensate density, molecular system mass, critical temperature, and number of charged residues along the sequence. Specifically, we use the reparameterization^124^ of the residue-resolution HPS model.^102^ We have recently shown that the HPS-cation-π reparameterization qualitatively reproduces the relative propensity of numerous RNA-binding proteins to phase separate under physiological conditions,^129^ as well as their RNA-concentration-dependent re-entrant phase behavior.^154−157^ Within this force field, hydrophobic and cation−π interactions are modeled through short-range pairwise potentials, and electrostatic interactions through a Yukawa/Debye–Hückel long-range potential. Additionally, bonded interactions between subsequent amino acids (or nucleotides) of the same protein (or RNA) are restrained by a harmonic potential. Moreover, within the HPS-cation-π force field the solvent is implicitly modeled by the screening length of the Yukawa/Debye–Hückel potential, which is tuned to reproduce protein phase behavior at physiological salt concentration (∼150 mM). All details regarding the force field parameters and simulation setups are provided in the Supporting Information.

The set of phase-separating biomolecules that we explore includes the following IDPs: DEAD-box helicase 4 (Ddx4),^158^ α-synuclein,^159^ microtubule-associated neuronal IDP Tau K18,^160^ the arginine-glycine rich-region of LAF-1 (LAF-1-RGG),^80^ and the low complex domains (LCD) of the heterogeneous nuclear ribonucleoprotein A1 (A-LCD-hnRNPA1),^13^ fused in sarcoma (FUS-LCD),^29^ and the TAR DNA-binding Protein of 43 kDa (TDP-43-LCD).^128^ Moreover, we investigate the viscoelastic behavior of 5 charge-matched complex coacervates (which can only phase-separate via heterotypic interactions):^100^ the proline-arginine 25-repeat dipeptide (PR_25_) in the presence of single-stranded disordered polyUridine RNA (polyU)^79^ of 50-nucleotides (polyU50) and 100-nucleotides length (polyU100); and three 50:50 binary mixtures of polyU and poly-Arginine (polyR)^59^ with different chain lengths, polyR50/polyU50, polyR50/polyU100, and polyR100/polyU100.

**Figure 2:**
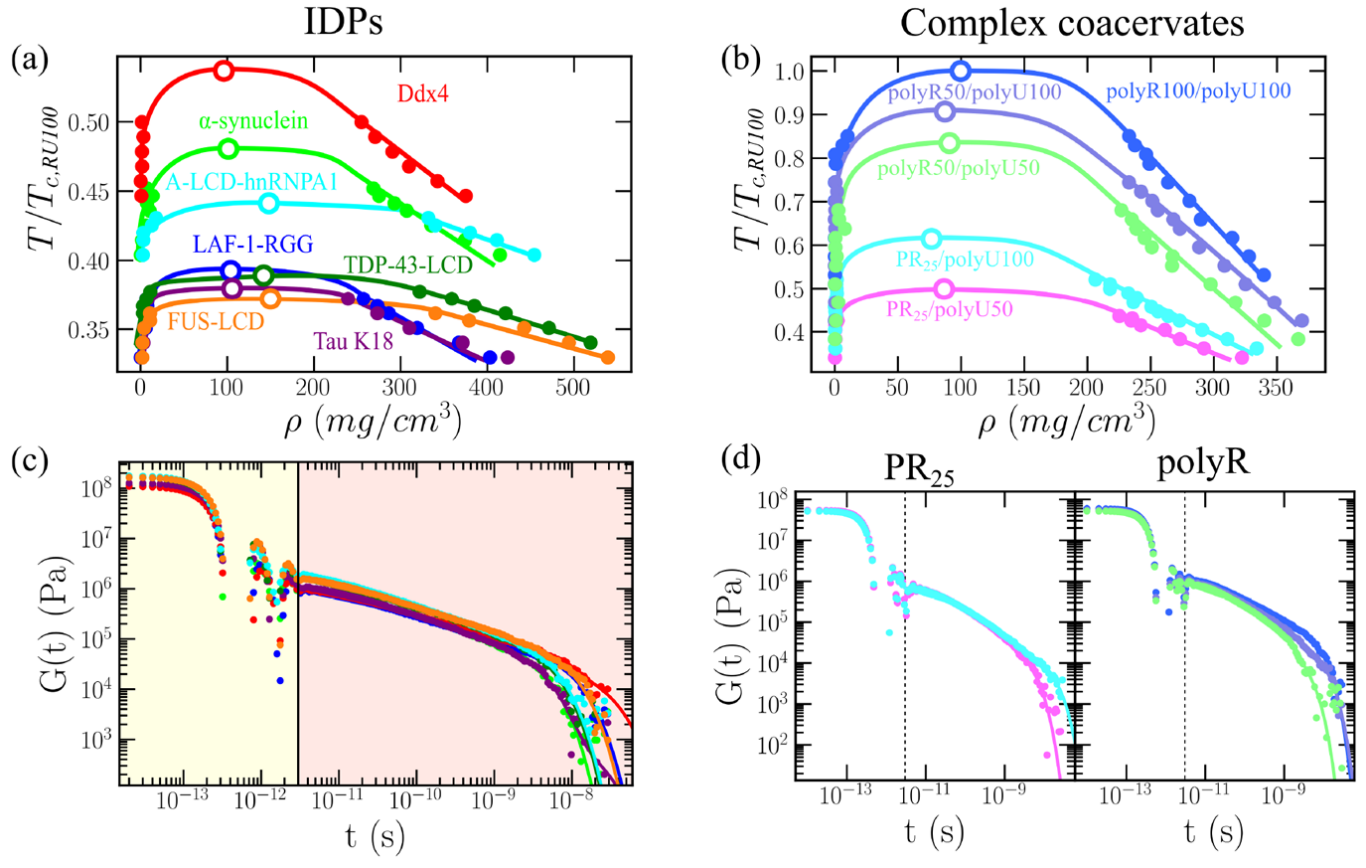
Phase diagram and shear stress relaxation modulus for a set of IDP/polyU phase-separated condensates. (a) Phase diagram in the *T*–ρ plane for Ddx4, α-synuclein, Tau K18, LAF-1-RGG, A-LCD-hnRNPA1, FUS-LCD, and TDP-43-LCD using the HPS-cation-π force field.^102,124^ (b) Phase diagram in the *T*–ρ plane for PR_25_/polyU50, PR_25_/polyU100, polyR50/polyU50, polyR50/polyU100, and polyR100/polyU100 using the HPS-cation-π force field.^102,124^ In both panels (a) and (b), filled symbols represent the coexistence densities obtained via DC simulations,^130^ while empty symbols depict the estimated critical points by means of the law of rectilinear diameters and critical exponents.^161^ Moreover, temperature has been renormalized by the critical temperature (*T*_*c*,RU100_ ≡ *T*_c,polyR100/polyU100_) of the system with highest *T*_*c*_, which is the charge-matched polyR100/polyU100. The statistical error is of the same order of the symbol size. (c) and (d) Shear stress relaxation modulus *G*(*t*) of the systems shown in panel (a) and (b) at *T*/*T*_*c*_^′^ ∼ 0.88 (referring *T*_*c*_^′^ to the critical temperature of each system) and at the bulk condensate density at such temperature. The vertical continuous (c) and dashed (d) lines separate the time scale corresponding to the computed term via numerical integration at short time scales and the part evaluated via the Maxwell modes fit at long time scales (eq 6).

We first evaluate the phase diagram (through DC simulations;^130^ see Supporting Information for further details) of the entire set of intrinsically disordered proteins (Figure 2(a)) and complex coacervates (Figure 2(b)). Remarkably, we find that the HPS-cation-π coarse-grained model qualitatively predicts the higher ability to phase separate for IDPs such as Ddx4^162^ or A-LCD-hnRNPA1^52,163^ compared to other low-complexity domains such as FUS-LCD^28,155^ or TDP-43-LCD,^164^ which require a higher protein saturation concentration in experiments. In contrast, the high critical saturation concentration of α-synuclein to undergo LLPS,^159^ which is similar to that of FUS-LCD^28,155^ is not qualitatively well predicted by the model. For the complex coacervates, we observe that for the same peptide/RNA length, i.e., PR_25_/polyU50 vs polyR50/polyU50, and PR_25_/polyU100 vs polyR50/polyU100, the polyR/polyU condensates always display higher ability to phase separate, in agreement with experimental *in vitro* findings.^59,79^ Furthermore, as expected, we also reproduce the higher ability to undergo LLPS as the length of the peptides and RNA increases in both families of complex coacervates (Figure 2(b)).

**Figure 3:**
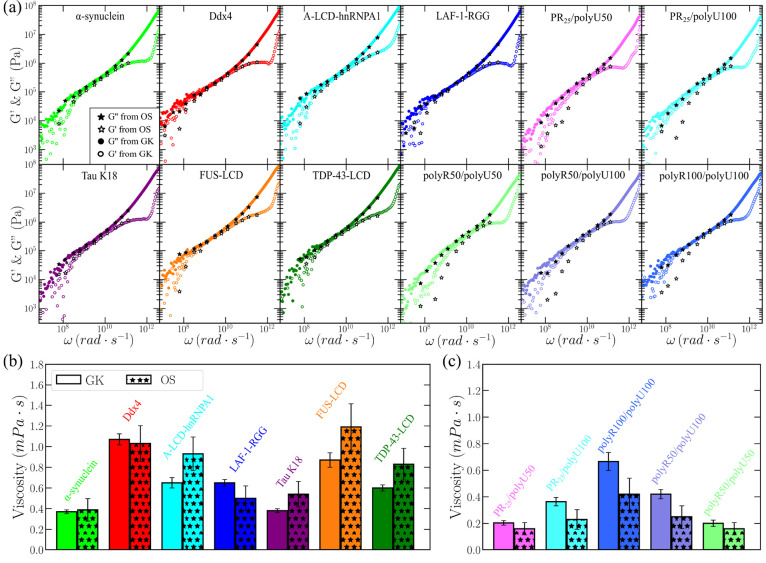
Condensate viscosity for different IDP and RNA complex coacervates evaluated through the GK relation and the oscillatory shear (OS) method. (a) Elastic (*G*′) and viscous (*G*″) moduli as a function of frequency (ω) from OS calculations at *T*/*T*_*c*_^′^ ∼ 0.88 (empty and filled stars respectively) and from GK method (empty and filled colored circles respectively) for different IDP condensates and complex coacervates as indicated in the legend. (b) Viscosity computed via the GK and OS methods for the distinct IDP condensates. (c) Viscosity obtained through the same two methods for the different complex coacervates.

Next, we evaluate the condensate viscosity for all systems employing the GK (Figure 2(c,d)) and OS (Figure 3(a)) methods. The conditions at which we undertake these calculations are at *T*/*T*_*c*_^′^ ∼ 0.88 (referring *T*_*c*_^′^ to the critical temperature of each system) and the condensate bulk density at such temperature. We choose such temperature to guarantee reasonable sampling across our simulations, and more realism provided by our protein/RNA condensate densities (excluding water due to implicit-solvent reasons) at such temperature usually being in the range from 0.2 to 0.4 g·cm^–3^; in reasonable agreement with experimental reported protein densities/concentrations within phase-separated condensates.^155,165,166^ Since in Figure 1(b) we show that the BT method only provides consistent estimations of η, independently of the probe bead size at relatively high temperatures or unless extremely long simulations are performed, for this set of biomolecular condensates we only carried out GK and OS calculations. In Figures 2(c,d) we show the shear stress relaxation modulus as a function of time for all IDPs and complex coacervates, respectively. In our simulations, all condensates are able to relax, i.e., the correlation function decays at long time scales, and therefore exhibit liquid-like behavior. Moreover, in Figure 3(a), we report the elastic (*G*′; empty stars) and viscous (*G*″; filled stars) moduli as a function of frequency from our OS calculations. The agreement in all cases for the entire regime of frequencies studied between OS and GK simulations is exceptional. The results of *G*′ and *G*″ from GK calculations have been obtained by applying the Fourier transform using the open source RepTate software.^167^ In accordance with the *G*(*t*) decays observed in Figure 2(c,d), the values of *G*″ are higher than *G*′ within the moderate frequency regime for all systems, further indicating that the viscoelastic behavior of the condensate is dominated by the loss modulus, e.g., liquid-like behavior, given that the stress is nearly in phase with the shear rate.

By integrating in time the shear stress relaxation modulus (eq 6) shown in Figure 2(c,d) for the distinct IDPs and complex coacervates, we can obtain the viscosity of the condensate. Moreover, taking the limit of eq 11 to very low frequencies, we can also evaluate η using the OS approach. In Figure 3(b,c), we report the viscosities obtained through the two methods for the set of IDPs and complex coacervates studied, respectively. It can be noted a fair agreement in the predicted viscosity between the OS and GK methods for most of the studied biomolecular condensates. Given that η is a magnitude that can dramatically vary (orders of magnitude) with small changes in protein intermolecular binding^29,59,73^ or in the applied thermodynamic conditions,^115,168^ the observation that for most condensates the predicted viscosity differs by less than a factor of 1/2, clearly indicates the robustness of our calculations. However, we acknowledge that the viscosity values from the GK calculations are likely more accurate than those using the OS method, since the former do not rely on the limit of *G*″/ω at very low frequencies, where the signal in the stress response is particularly low.^116^

**Figure 4:**
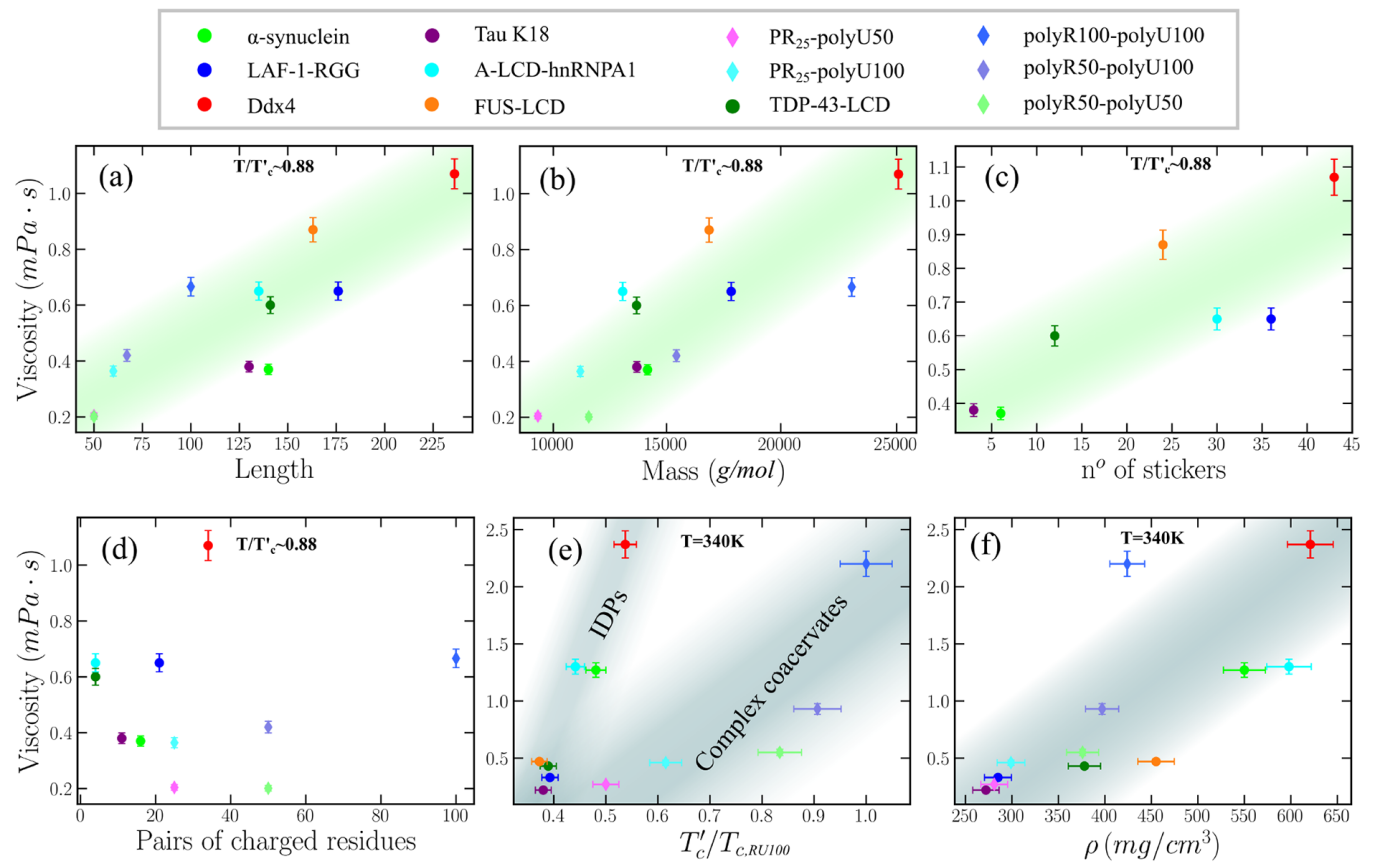
Correlation between condensate viscosity (obtained via the GK method) and chain length (a), molecular weight (b), number of stickers (Y, F, and R) across the sequence (c), and number of charged residue pairs of opposite charge (d) at constant *T*/*T*_*c*_^′^ ∼ 0.88. In contrast, the correlation between viscosity and condensate critical temperature (e) and density (f) is plotted for a constant *T* = 340 K. In complex coacervates, we take the average chain length and molecular weight of the two cognate molecules. In panel (e), we note that each critical temperature has been renormalized by the highest critical temperature of the studied set (*T*_*c*,RU100_, which corresponds to that of polyR100/polyU100). Since the complex coacervates by construction do not contain aromatic residues, their results have not been considered for the correlation shown in panel (c).

Interestingly, from the results shown in Figure 3(b,c) at *T*/*T*_*c*_^′^ ∼ 0.88, we find a clear correlation between viscosity and protein/RNA length, defined as the number of amino acids or nucleotides in the macromolecule (Figure 4(a)). As the length of the IDP or RNA chain increases, the viscosity of the phase-separated condensates also augments, and, therefore, the required computational cost to determine η. Indeed, the correlation seems to be linear, suggesting a behavior characteristic of the sticky Rouse model developed by Rubinstein,^169,170^ considering that the concentration of proteins inside the condensate is sufficiently high so that the strands between stickers overlap. Therefore, the viscoelastic properties of these condensates cannot be described by reptation^146^ or sticky reptation dynamics^169,171^ because the density of the condensates and the molecular weight of the proteins are not high enough to consider the effect of entanglements. As can be seen in Figure 4(b), the same correlation holds for the protein/RNA molecular mass (Figure 4(b)) since the increase in length directly impacts the molecular weight. However, when plotting η against condensate density at *T*/*T*_*c*_^′^ ∼ 0.88 for all systems, we do not observe a clear trend (Figure S3(e)). That is a striking result given that larger densities or packing fractions should lead to higher viscosity values.^129,168^ Nevertheless, we identify cases such as Ddx4 in which despite presenting a low condensate density, its viscosity is the highest (in correspondence with its length). On the other hand, A-LCD-hnRNPA1 has a moderate length, i.e., 135 residues, its condensates are the most dense of the set, and its viscosity is just moderate (Figure S3(e)). This suggests that there are other factors, such as the number and strength of stickers^169^ or the charge density, that may have a stronger effect than density on the viscosity of the condensate. We also interrogate the correlation between η measured at *T*/*T*_*c*_^′^ ∼ 0.88 and the relative critical temperature of each system, i.e., ability to undergo LLPS in our model, which is directly inverse to the protein saturation concentration.^162^ As shown in Figure S3(d), there is no clear evidence, according to our simulations, that systems with higher critical temperature should present higher viscosity as long as the conditions at which η is determined are equidistant to *T*_*c*_^′^ for each system. Nonetheless, when our measurements are carried out at constant *T* = 340 K for all the systems instead of *T*/*T*_*c*_^′^ (Figure 4(e)), a clear dependence is observed between condensate viscosity and critical temperature for the studied set of IDPs and complex coacervates. We hypothesize that this correlation might be also strongly dependent on the type of interactions promoting LLPS, being mostly of electrostatic nature for the complex coacervates, and a combination of hydrophobic, cation−π, π–π, and electrostatic interactions for the IDP set. Furthermore, we find that for a constant temperature (i.e., *T* = 340 K) a strong correlation between condensate viscosity and density arises (Figure 4(f)) in contrast with the results shown at constant *T*/*T*_*c*_^′^ for all systems (Figure S3(e)).

We also analyze the correlation of viscosity with the sequence composition across the different studied IDPs (Figure 4(c,d)). First, we focus on how η is related to the number of stickers and spacers along the different sequences. The framework of stickers and spacers for protein phase separation represents multivalent proteins as heteropolymers made of stickers (i.e., binding sites for associative interactions) and spacers (regions in between stickers).^92,172,173^ Following ref.,^126^ we have considered tyrosine (Y), phenylalanine (F) as main stickers, arginine (R) as a context-dependent sticker, and the rest of the amino acids as spacers. In Figure 4(c), we show how the viscosity is proportional to the sticker abundance in the different IDPs studied at constant *T*/*T*_*c*_^′^, in agreement with the predictions of the sticky Rouse theory.^169^ This is an expected result since stickers, due to their stronger intermolecular binding, act as amino acids with an effectively higher friction coefficient, which slows the conformational relaxation of the biomolecules and, thus, increases the viscosity of the condensate. The trend only partially deviates for the case of LAF-1-RGG due to the extremely high abundance of R, which is a context-dependent sticker, and in the absence of aromatic residues, R self-repulsion dominates.^126^ Importantly, if we only consider the aromatic residues as stickers, then the correlation between sticker abundance and viscosity becomes poorer than when we also include R as sticker (Figure S5), highlighting the role of R as a sticker when there is high abundance of aromatics (Figure 4(c)). When condensate viscosity is plotted against the protein sticker abundance for a constant *T* instead of *T*/*T*_*c*_^′^, a poorer correlation is found (Figure S4(c)). We hypothesize that the reason behind this behavior might be that at constant *T*, the significant different densities between distinct phase-separating condensates (Figure 4(f)), in turn regulated by a complex amalgam of sequence features, including the sticker abundance, is mostly controlling the condensate viscosity. Since our simulations are effectively describing the protein phase behavior at physiological salt concentration, where electrostatic interactions are known to play a key role in sustaining LLPS,^174^ we now ask whether a correlation can be found between droplet viscosity and the abundance of charged residues of distinct sign, i.e., total number of pair residues with opposite charge. We show that, very mildly, viscosity might be proportional to the number of charged residues of opposite sign along the studied IDP sequences: at constant *T*/*T*_*c*_^′^ (Figure 4(d)) and at constant *T* (Figure S4(f)). In this line, for both A-LCD-hnRNPA1 and TDP-43-LCD, the number of pair residues with opposite charge is significantly low, and their condensate viscosity is still moderate, showing a much better correlation with the IDP length/molecular weight or the number of stickers throughout the sequence than with the number of charged residues. Moreover, the role of charge patterning, as recently shown by refs (80), (104), and (175), can also critically modulate the stability and viscosity of the condensates. Hence, finding a correlation between condensate viscosity and the number of charged residues of opposite sign along the sequences is not trivial at all.

It is important to note that our viscosity results presented in Figures 3 and 4 generally underestimate the experimental values of η for *in vitro* phase-separating condensates.^140,176^ The coarse-grained nature of the HPS-cation-π force field in which amino acids and nucleotides are represented by spherical beads in combination with an implicit solvent model, importantly speeds up the system dynamics and leads to an underestimation of approximately 2–3 orders of magnitude in η. However, the computational efficiency of the model also enables this type of calculations for phase-separated condensates formed by hundreds of protein replicas.^129^ Importantly, with our calculations we recover the experimental observation that increasing polyR and polyU length significantly enhances condensate viscosity, as well as the key role of arginine−uridine interactions in triggering LLPS and increasing viscosity.^59^ As shown in Figure 3(c), arginine–uridine interactions are much more relevant than those of proline–proline, proline–arginine or proline–uridine in regulating condensate viscosity and stability. Furthermore, we recapitulate the observation that within the experimental uncertainty, the viscosity of LAF-1-RGG,^176^ TDP-43-LCD,^177^ and FUS-LCD^115^ condensates (before maturation) is approximately of the same order. Additionally, it has been reported that Ddx4 inside phase-separated condensates possess an extremely low translational diffusion;^158^ our results from Figure 4 also qualitatively suggests this behavior. For Tau K18 and α-synuclein condensates, to the best of our knowledge, there are no available results for η. However, our results for Tau K18 support that phase separation can be experimentally observed only in the presence of molecular crowders, or through complex coacervation with RNA^178^ due to its low abundance of aromatic residues (as shown in Figure 4(c), maroon circle). Hence, experimental observations such as those from ref (178) may justify the low viscosity (Figure 3(b)) and critical temperature (Figure 2(a)) obtained for Tau K18 condensates.

In summary, although the viscosities predicted by sequence-dependent protein/RNA coarse-grained models^101,124,125,179^ cannot quantitatively match experimental results,^140^ they can provide valuable qualitative trends on how the viscoelasitc properties of a given condensate may change upon variations on the thermodynamic conditions,^115^ post-translational modifications,^180^ mutations,^49^ or addition of different cognate molecules.^59^ Therefore, establishing robust methodologies to evaluate viscosity via computer simulations can be of great relevance to envision possible strategies to regulate such critical magnitude in the condensate function.

### Maturation of Protein Condensates Can Be Unequivocally Tracked by the OS and GK Techniques

In this section, we investigate the progressive rigidification of phase-separated condensates due to the gradual accumulation of interprotein structural transitions over time.^13,113,127^ It has been recently shown, both experimentally and computationally, that the interaction landscape of proteins can be significantly transformed by structural transitions.^49,72,74,75,113^ The low complexity domains (LCD) of many naturally occurring phase-separating proteins, including FUS,^48^ TDP-43,^49,128^ or hnRNPA1^13,127^ among many others,^113,181^ contain short regions termed Low-complexity Aromatic-Rich Kinked Segments (LARKS), which are prone to forming interprotein β-sheets in environments of high protein concentration.^13,115,182^ Although these proteins can form liquid-like condensates, depending on the conditions, i.e., temperature^115^ or concentration,^113^ they can also transition into hydrogels over time.^46,183,184^ Hence, interprotein structural transitions have been proposed to trigger transient solidification of, otherwise, liquid-like condensates.^44,185^ Importantly, misregulation of biomolecular condensates into solid-like aggregates is associated with the onset of several neurodegenerative diseases.^35,186^ Therefore, motivated by these observations, here we explore by means of the GK and OS techniques the impact of transient accumulation of β-sheet fibrils in the viscoelastic properties of phase-separated condensates formed by LARKS-containing LCDs of FUS, TDP-43, and hnRNPA1.

For these simulations, we employ an aging dynamical algorithm recently developed by us^72,74^ to describe the nonequilibrium process of condensate maturation due to interpeptide β-sheet formation. Coupled to the HPS-cation-π residue-resolution model,^102,111,124^ our dynamical algorithm approximates the condensate maturation process by considering the atomistic implications, i.e., nonconservative strengthening of interprotein binding and local protein rigidification,^73^ of the gradual and irreversible accumulation of interprotein β-sheet structures in a time-dependent manner, and as a function of the local protein density within phase-separated condensates. In practice, our dynamical algorithm triggers transitions from disordered LARKS to interprotein structured β-sheets when the central C_α_ bead of a LARKS is in close contact (within a cutoff distance of ∼8 Å) with three other LARKS of neighboring proteins.^48,49,113^ Therefore, every 100 simulation time steps, our algorithm evaluates whether the conditions around each fully disordered LARKS are favorable to undergo an “effective” disorder-to-order β-sheet transition. In our model, the structural transition is recapitulated by enhancing the interaction strength of four LARKS–LARKS peptides based on the results of our atomistic potential-of-mean force simulations.^72,74,75^ In the atomistic simulations, we estimate the binding free energy difference between disordered interacting LARKS peptides, and interacting LARKS peptides that are forming interprotein cross β-sheets. We estimate these changes for the three FUS LARKS within the LCD (_37_SYSGYS_42_, _54_SYSSYGQS_61_, and _77_STGGYG_82_),^48,72^ the A-LCD-hnRNPA1 LARKS (_58_GYNGFG_63_),^74,113^ and that of TDP-43^75^ (_58_NFGAFS_63_; also located in the protein LCD)^49^. Therefore, by employing the HPS-cation-π model coupled to our dynamical aging algorithm, we can effectively investigate the viscoelastic behavior of FUS-LCD, A-LCD-hnRNPA1, and TDP-43-LCD condensates prior and postmaturation. Technical details on the aging dynamical algorithm, the local order parameter driving structural transitions, and the structured interaction parameters of the coarse-grained model are provided in the Supporting Information.

**Figure 5:**
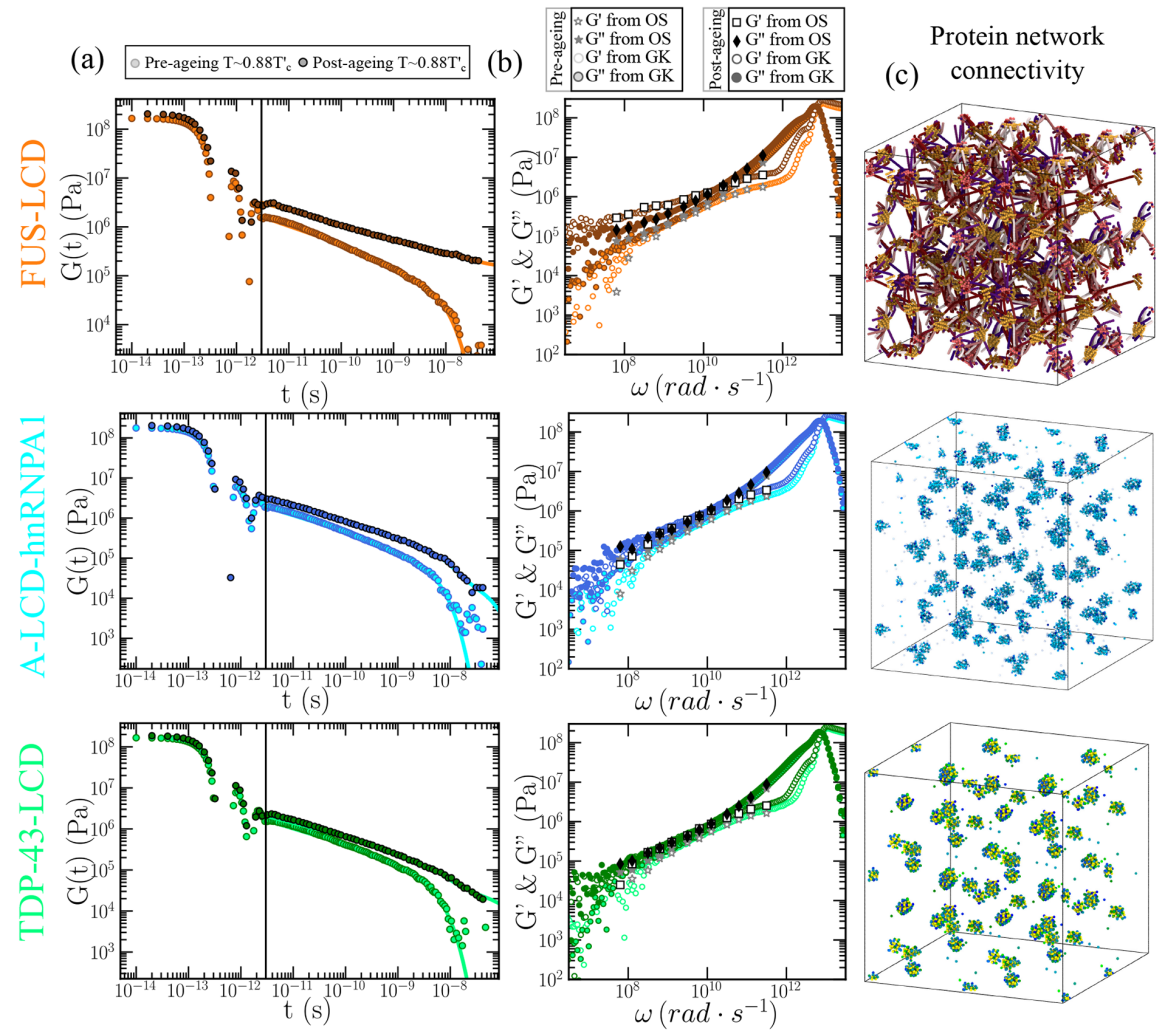
Viscoelasticity measurements and condensate network connectivity analysis of FUS-LCD, A-LCD-hnRNPA1, and TDP-43-LCD aged protein condensates. (a) Shear stress relaxation modulus of FUS-LCD (top), A-LCD-hnRNPA1 (middle), and TDP-43-LCD (bottom) condensates at *T* ∼ 0.88*T*_*c*_^′^ prior maturation (light colors; reference model HPS-cation-π)^102,124^ and after 400 ns of maturation (dynamical aging model).^74^ (b) Elastic modulus *G*′ (empty symbols) and loss modulus *G*″ (filled symbols) of FUS-LCD (top), A-LCD-hnRNPA1 (middle), and TDP-43-LCD (bottom) condensates from computational oscillatory shear simulations prior (stars) and after maturation (squares and diamonds). *G*′ (empty circles) and *G*″ (filled circles) evaluated through the Fourier transform of *G*(*t*) via the GK relation pre-aging (light colors) and post-aging (dark colors) are also included. (c) Network connectivity of aged FUS-LCD (top), A-LCD-hnRNPA1 (middle), and TDP-43-LCD (bottom) condensates at *T* ∼ 0.88*T*_*c*_^′^ after 400 ns of maturation computed using the primitive path analysis.

We start by applying the GK and OS methods to phase-separated condensates under bulk conditions of FUS-LCD, A-LCD-hnRNPA1, and TDP-43-LCD at *T* ∼ 0.88 *T*_*c*_^′^ prior maturation (light brown, blue, and green circles respectively in Figure 5(a)). Please note that the critical temperature is barely affected by the maturation of the condensate, as demonstrated in our previous study.^74^ As can be seen, *G*(*t*) prior maturation decays sharply in the terminal region evidencing liquid-like behavior and full relaxation of the condensates. Moreover, we also apply the OS method to evaluate the loss and viscous moduli as a function of frequency for these condensates prior-aging (stars in Figure 5(b)). We find an exceptional agreement between *G*′ and *G*″ as a function of frequency using both OS and GK techniques for FUS-LCD, A-LCD-hnRNPA1, and TDP-43-LCD condensates. Then, we activate the aging dynamical algorithm^72,74,75^ and perform 0.4 μs simulations under bulk condensate conditions to allow interprotein structural transitions to accumulate over time. In Figure S2, we show the time-evolution of structural transitions driven by high-density protein fluctuations leading to interprotein β-sheet domains within the condensates. For all cases, within the tested maturation time, the percentage of LARKS forming interprotein β-sheet domains is higher than 75% (Figure S2; please note that the faster dynamics of the protein model also increases the condensate maturation rate as discussed in ref (74)).

We now evaluate *G*(*t*) for the FUS-LCD, A-LCD-hnRNPA1, and TDP-43-LCD aged condensates after 0.4 μs of maturation time (Figure 5(a); darker circles). Remarkably, we find that for all condensates the observed decay in *G*(*t*) prior maturation is no longer present (light circles in Figure 5(a)). Irrespective of the protein, aging increases significantly the values of the shear stress relaxation modulus; hence, suggesting a much higher viscosity for aged condensates than for their pre-aged counterparts. Nevertheless, when looking more closely at the time-dependent behavior of *G*(*t*), we observe significantly distinct curves for the different protein LCDs. While for both A-LCD-hnRNPA1 and TDP-43-LCD the continuous decay of *G*(*t*) suggests that aged condensates will present liquid-like behavior at very long time scales (high-viscous liquids), in FUS-LCD *G*(*t*) falls into a persistent plateau with no hints of decaying at comparable time scales, and yielding infinite viscosity values, i.e., nondiffusive behavior, characteristic of a gel-like state as recently experimentally reported for FUS^29^ and FUS-LCD^115^ condensates. The fundamental difference for FUS-LCD condensates exhibiting gelation upon condensate maturation is the presence of three separate LARKS along its sequence. At least two multivalent or three monovalent anchoring points per molecule are necessary for a system to completely gelate.^116^ Thus, the strong gel-like behavior exhibited by FUS-LCD condensates is not expected to occur in A-LCD-hnRNPA1 or TDP-43-LCD with only a single LARKS since, strictly speaking, the intermolecular network of β-sheets would not be able to fully percolate unless another anchoring domain along the sequence could also establish strengthened interprotein binding, i.e., due to a sequence mutation or a post-translational modification.^49^ According to our *G*(*t*) results shown in Figure 5(a), both A-LCD-hnRNPA1 and TDP-43-LCD condensates exhibit very high-viscous behavior after maturation.

In Figure 5(b), we also plot *G*′ and *G*″ postaging for FUS-LCD, A-LCD-hnRNPA1, and TDP-43-LCD condensates evaluated through both OS and GK methods. In agreement with the results shown in Figure 5(a), we find that for FUS-LCD condensates, *G*′ upon maturation is higher than *G*″, thus indicating gel-like behavior. In contrast, in matured A-LCD-hnRNPA1 and TDP-43-LCD condensates, the viscous modulus is higher than the elastic one, hence confirming the high-viscous liquid-like behavior pinpointed from *G*(*t*) calculations (Figure 5(a)) for these condensates.

To further characterize the structure and topology of the aged condensates in terms of the β-sheet intermolecular network, we apply a modification of the primitive path analysis (PPA) algorithm.^187,188^ In our PPA calculations, we consider the β-sheet binding fixed in space, the bond interaction is modified to have an equilibrium bond length of 0 nm, and the intramolecular excluded volume is set to zero. The algorithm then minimizes the contour length of the protein strands that connect the different LARKS regions while preserving the topology of the underlying network. Furthermore, we replicate the system in all directions of space to better visualize the extension of the network connectivity beyond the periodic boundary conditions of the simulation box. At the end of the minimization, this method allows the visualization of the network connectivity generated by interprotein β-sheet clusters (Figure 5(c)). For FUS-LCD matured condensates (Figure 5(c); top panel), we find an elastically percolated network of protein strands that contributes to the formation of a rubbery plateau in *G*(*t*) (as shown in Figure 5(a); top panel). This β-sheet percolated network also explains the higher value of *G*′ respect to *G*″ upon maturation (Figure 5(b); top panel). On the other hand, in mature A-LCD-hnRNPA1 and TDP-43-LCD condensates, proteins form isolated β-sheet clusters (Figure 5(c); middle and bottom panels, respectively). These results from the PPA analysis are also in agreement with *G*(*t*) decaying to zero at much longer time scales, i.e., showing a higher viscosity but not a rubbery plateau as FUS-LCD, and with the viscous modulus being higher than the elastic one (Figure 5(a,b); middle and bottom panels, respectively).

Our results from Figure 5 are fully consistent with recent experimental observations of FUS-LCD condensates where an increase in the β-sheet content has been associated with protein dynamical arrest within phase-separated condensates.^115^ Progressive kinetic arrest through the emergence of long-lived intermolecular interactions giving rise to β-sheet percolated networks (Figure 5(c)) is also consistent with the experimental observation of reversible hydrogels in LARKS-containing RNA-binding proteins after maturation (such as TDP-43 or FUS)^48,49,113^ that can be dissolved with heat, and where a high percentage of β-sheet content has been found.^115^ Furthermore, our results help to explain the recognized asphericity of aged condensates^29,189^ and the emergence of irregular morphologies caused by nonergodic droplet coalescence^73,115^ reported for LCD-containing proteins such as hnRNPA1,^12^ FUS,^29^ TDP-43,^190^ or NUP-98.^181^ Remarkably, the progressive kinetic arrest of proteins within droplets in FUS (full-sequence) in combination with a severe imbalance in the intermolecular forces has been shown to drive single-component condensates to display multiphase architectures upon maturation^72,191^ or upon phosphorylation.^192^

## Discussion and Conclusions

**Figure 6:**
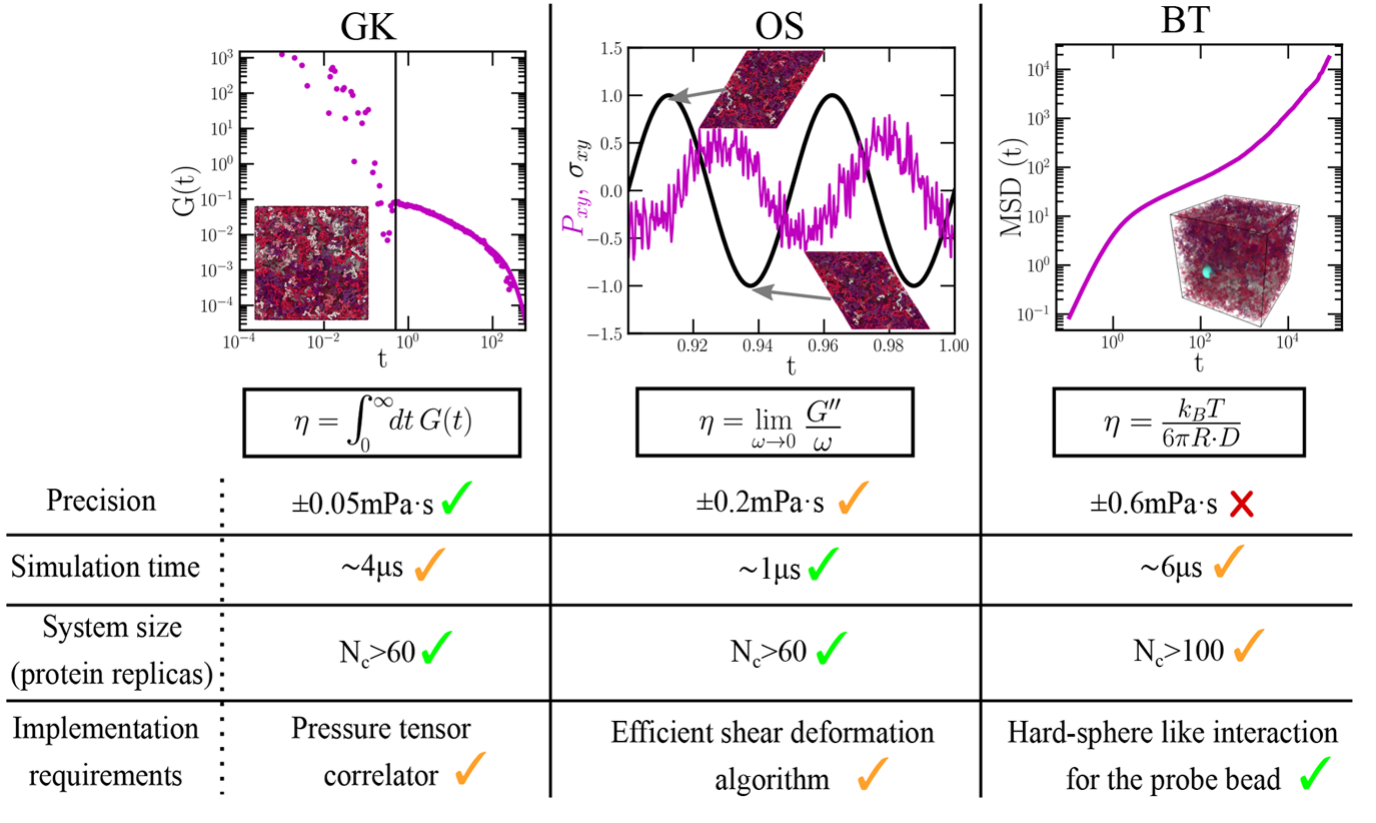
Comparison of the three different employed computational techniques to evaluate viscosity in phase-separated condensates. In the top panels we show the following: (left) decay over time of the shear stress relaxation modulus for computing η through the GK method; (middle) applied shear deformation (σ_*xy*_; black curve) and stress response (*P*_*xy*_; purple curve) as a function of time evaluated through the oscillatory shear method; (right) mean squared displacement of the probe bead (blue particle in the inset) to determine η through the Stokes–Einstein relation. Importantly, we note that GK and OS calculations do not depend on the system size as long as protein self-interactions are avoided through the periodic boundary conditions, whereas in BT simulations, such conditions might not be enough to prevent finite system-size effects in cases where the probe bead radius is greater than the protein radius of gyration. The specified data in the table applies for IDPs as those studied in this work.

In this work, we have applied different computational techniques to characterize the viscoelastic behavior of biomolecular condensates formed by proteins and RNA, and modeled through coarse-grained potentials of different resolution. First, by means of a simple coarse-grained model for studying IDP phase separation,^99^ we have tested the validity, accuracy, and computational performance of three numerical methods to evaluate the viscosity of condensates. These methods are the shear stress relaxation modulus integration (GK),^116,117^ the oscillatory shear (OS) technique,^118,119^ and the bead tracking (BT) method.^29,120−122^ In Figure 6, we summarize their different advantages and drawbacks in terms of precision, required simulation time, system size, and major computational implementation requirements. Importantly, we find that the GK method is the most accurate approach to compute η since it does not require the extrapolation of *G*″/ω to the limit of ω → 0 (where the stress response signal is weak, as in the OS method) or the need of extremely long simulation time scales to avoid probe bead size-dependence (Figure 1(b)). On the other hand, the OS approach possesses the advantage that the required simulation time scale to obtain reasonable estimates of η is approximately 4 and 6 times lower than the GK and BT methods, respectively. Nevertheless, it relies on the implementation of a sophisticated shear deformation algorithm to perform oscillatory shear (Figure 6). In terms of system size, while the three methods require a reasonable amount of protein replicas to avoid finite-size effects (due to protein self-interactions through the periodic boundary conditions), we note that the BT method may still demand even larger system sizes for cases where the probe radius is of the order or greater than the protein radius of gyration. However, the key advantage of this method is the simplicity of its implementation, which only requires the insertion of a probe bead with a hard sphere-like interaction with the surrounding media and the calculation of its mean squared displacement within the diffusive regime. Therefore, despite the fact that each technique has its own pros and cons (Figure 6), the GK method presents the highest overall performance in terms of accuracy, implementation, and computational feasibility.

Then, after having tested these different approaches for a simple model of IDP LLPS, we have applied the GK and OS techniques for determining the condensate viscosity of a set of 7 different IDPs and 5 peptide/RNA complex coacervates using a sequence-dependent high-resolution coarse-grained model^101,124,125^ (Figures 2 and 3). We find a reasonable agreement in the predicted viscosity between both techniques for all these systems (Figure 3(b,c)) despite the weak stress signal of the OS method at low frequencies, which slightly hampers the calculation of η (especially for systems with low density and long chains; i.e., polyR100/polyU100). Such agreement between both techniques can be especially noticed when plotting *G*′ and *G*″ vs a wide range of frequencies for all systems, as shown in Figure 3(a).

We also identify through our simulations a clear correlation of condensate viscosity with IDP/RNA length, molecular weight, and the number of LLPS stickers across the protein sequence (Figure 4) when we compare all the systems at the same relative temperature with respect to the critical one. Importantly, within the stickers and spacers framework,^92,172,173^ we find the best correlation when considering tyrosine, phenylalanine, and arginine as LLPS stickers^126^ than when only including aromatic residues.^193^ On the contrary, when performing our calculations at constant *T* instead of constant *T*/*T*_*c*_^′^, we find that IDPs and complex coacervates with higher critical temperature display higher viscosity. Similarly, higher condensate densities correlate with higher viscosities when comparing at a fixed temperature (Figure 4(f)). Furthermore, since our simulations effectively describe the protein phase behavior at physiological salt concentration (where electrostatic interactions are known to play a key role in sustaining LLPS),^174^ we also test a possible correlation between condensate viscosity and the abundance of pairs of charged residues of distinct sign. However, a very mild trend, if any, is observed for the studied IDPs and complex coacervates as a function of the number of residue pairs with opposite charge (at least when no charge patterning is considered; Figure 4(d)).

Last, we have investigated by means of the GK and OS methods the progressive maturation through β-sheet accumulation of three of the most relevant protein low-complexity domains related to the onset of ALS and FTD diseases:^32,35^ A-LCD-hnRNPA1,^13,113,127^ FUS-LCD,^48^ and TDP-43-LCD.^49,128^ We find that both techniques predict the transition from condensate liquid-like behavior to partially (A-LCD-hnRNPA1 and TDP-43-LCD) or fully (FUS-LCD) kinetically trapped states once an intermolecular β-sheet network has grown through the condensate, thus hindering protein self-diffusion at moderate time scales. Remarkably, the (experimentally reported^115^) emergence of gel-like behavior in FUS-LCD condensates, due to an increase in the β-sheet content, can be straightforwardly identified through the OS and GK methods by the higher values of *G*′ with respect to *G*″ at moderately low frequencies (Figure 5(b); top panel). Moreover, the behavior of *G*(*t*) falling into a persistent plateau (Figure 5(a); top panel) corroborates the gel-like behavior of aged FUS-LCD condensates with respect to matured A-LCD-hnRNPA1 and TDP-43-LCD droplets still presenting high-viscous liquid-like behavior with much longer relaxation time scales than their preaged counterparts (Figure 5(a); middle and bottom panels, respectively). These results are also confirmed by PPA calculations revealing the underlying interprotein β-sheet network emerged upon maturation (Figure 5(c)). Taken together, our study provides a compilation of modeling rheological techniques to assess the viscoelastic properties of biomolecular condensates and link them to the behavior of their constituent biomolecules.

## Data Availability

The most representative simulation inputs and LAMMPS scripts for the systems studied here can be found at https://zenodo.org/badge/latestdoi/626526745.

## Supporting Information Available

Additional supporting material is included in the associated Supporting Information file. We include a description of the computational models use for the simple CG model and the HPS+cation−π model to simulate proteins, details about the calculation of the phase diagrams, the sequences of the proteins studied in this work, technical details about system size and the three methods studied here, a detail description of the dynamical algorithm to mimic protein structural transitions (including a table with the used parameters), a description of the PPA technique to study the protein network connectivity, plots of the viscosity using different bead radii for the BT technique, the evolution of cross-β-sheet transitions for the three studied proteins, and additional perspectives on the correlation of viscosity with other magnitudes (PDF)
